# Transthyretin and BRICHOS: The Paradox of Amyloidogenic Proteins with Anti-Amyloidogenic Activity for Aβ in the Central Nervous System

**DOI:** 10.3389/fnins.2017.00119

**Published:** 2017-03-15

**Authors:** Joel N. Buxbaum, Jan Johansson

**Affiliations:** ^1^Department of Molecular and Experimental Medicine, The Scripps Research InstituteLa Jolla, CA, USA; ^2^Scintillon InstituteSan Diego, CA, USA; ^3^Division of Neurogeriatrics, Department of Neurobiology, Care Sciences, and Society (NVS), Center for Alzheimer Research, Karolinska InstitutetHuddinge, Sweden

**Keywords:** transthyretin, BRICHOS, Alzheimer's disease, amyloidosis, neurodegeneration, protein-protein interactions, chaperones

## Abstract

Amyloid fibrils are physiologically insoluble biophysically specific β-sheet rich structures formed by the aggregation of misfolded proteins. *In vivo* tissue amyloid formation is responsible for more than 30 different disease states in humans and other mammals. One of these, Alzheimer's disease (AD), is the most common form of human dementia for which there is currently no definitive treatment. Amyloid fibril formation by the amyloid β-peptide (Aβ) is considered to be an underlying cause of AD, and strategies designed to reduce Aβ production and/or its toxic effects are being extensively investigated in both laboratory and clinical settings. Transthyretin (TTR) and proteins containing a BRICHOS domain are etiologically associated with specific amyloid diseases in the CNS and other organs. Nonetheless, it has been observed that TTR and BRICHOS structures are efficient inhibitors of Aβ fibril formation and toxicity *in vitro* and *in vivo*, raising the possibility that some amyloidogenic proteins, or their precursors, possess properties that may be harnessed for combating AD and other amyloidoses. Herein, we review properties of TTR and the BRICHOS domain and discuss how their abilities to interfere with amyloid formation may be employed in the development of novel treatments for AD.

## Introduction

The amyloidoses are a set of human diseases (and their animal models) in which the precursors, synthesized as soluble proteins, aggregate and become insoluble under physiologic conditions. They ultimately form ultrastructural non-branching fibrils 10 nm in diameter with a characteristic cross-β sheet structure on x-ray diffraction analysis (Glenner, 1980). In tissues the fibrillar aggregates are seen as extracellular plaque-like structures in the affected organs, which bind the dye Congo red displaying green birefringence under polarized light (Linke, 2006). Amorphous pre-fibrillar or “off-pathway” aggregates may be present in the same tissues. Thus far 36 proteins have been noted to be associated with local or systemic amyloid deposition (Sipe et al., 2016).

In recent years with the ability to make large amounts of recombinant proteins from virtually any source, it has been noted that many proteins unrelated to disease will form fibrils under amyloid forming conditions and generate oligomeric species that are cytotoxic *in vitro* (Bucciantini et al., 2002). In mammalian cells several examples of physiologically functional amyloids have been described (Fowler et al., 2005; Maji et al., 2009). Approximately ~0.2% of all human proteins (36/20,000) form disease associated or functional amyloids. Further, in prokaryotes amyloid structures function as critical elements in biofilm formation (Blanco et al., 2012). One explanation for the apparent propensity of only some proteins to be fibrillogenic *in vivo* is that potentially amyloidogenic protein domains are “self-chaperoned” in the context of their intact native conformations. Perhaps evolution has recognized both the functional usefulness of sequences that can lead to amyloid formation and the risk of aggregation *in vivo* and embedded them in structures that do not allow the aggregation prone domains to form the homotypic interactions (“stearic zippers”) required for fibrillogenesis under physiologic conditions (Goldschmidt et al., 2010). Alternatively it may be that all proteins have the capacity to form amyloid under the appropriate conditions with those conditions rarely found in biology (Dobson, 1999). In eukaryotes, apart from so-called “normal” amyloids, it is rare for wild type proteins to form amyloid *in vivo* unless they undergo some modification, e.g., cleavage, as in the case of the Aβ protein precursor (AβPP) of Alzheimer's disease (AD) or the serum amyloid A (SAA) protein precursor in inflammation associated amyloidosis (Haass and Selkoe, 1993; Kluve-Beckerman et al., 2002). More likely is the occurrence of variants encoded by a mutation in the germline gene, as is the case with transthyretin (TTR) and other precursors responsible for human autosomal dominant hereditary amyloidoses (Rowczenio et al., 2014). It has also been argued that evolution has resulted in cells/organisms constraining the synthesis of some potentially amyloidogenic molecules to ensure that quantitatively they do not exceed the critical concentrations sufficient to nucleate the aggregation process in the presence of adequate cellular chaperone activity (Tartaglia et al., 2007).

In humans, while many of the amyloidoses are systemic in distribution, a disproportionate number are represented in the neurodegenerative diseases associated with aging. The prototype is AD, a disorder characterized by progressive memory loss and behavioral changes. Current thinking, based on genetic, biochemical and *in vivo* observations, favors the notion that cleavage fragments of the normal single pass transmembrane molecule AβPP, i.e., Aβ_1–38–43_, which are aggregation prone, form oligomers, which can be shown to be cytotoxic in tissue culture and synaptotoxic in hippocampal slices (Selkoe and Hardy, 2016). It is not yet clear if the toxic oligomers are on the same folding pathway as the fibrils found in the Congophilic deposits in the brain or whether the fibrils are a less toxic form of aggregate (Wu et al., 2013). The sequence of AβPP cleavage followed by aggregation of Aβ fragments is causal in the rare autosomal dominant forms of AD and is likely to participate in the pathogenesis of the sporadic disease, but may not be the sole etiology in the latter. In both forms there appears to be a multiplicity of contemporaneous or downstream events involving other cell types (microglia, astrocytes) and proteins, particularly Tau, which contribute to the development of the characteristic dementia.

During the last decade studies in transgenic models of human Aβ deposition have shown that many human genes and the silencing of a number of mouse genes may have profound impacts on the pathogenesis of the AD-like changes seen in the murine models and have suggested roles for these molecules in inhibiting or facilitating the process of amyloidogenesis *in vivo*. The salutary effects of two such genes (*TTR* and *ITMB2*) were quite unexpected since by themselves both protein products were clearly amyloidogenic and responsible for distinct forms of clinically relevant human amyloidosis.

Wild type and mutant forms of human TTR cause a spectrum of human systemic amyloid syndromes including Familial Amyloidotic Cardiomyopathy (FAC), Familial Amyloidotic Polyneuropathy (FAP) and Senile Systemic Amyloidosis (ATTRwt). Mutant forms of *ITM2B*, encoding the BRICHOS (see below) domain containing Bri2 protein are etiologic in Familial British Dementia and Familial Danish Dementia, while mutations in the gene (*SFTPC*) encoding another protein with a BRICHOS domain, lung surfactant C precursor (proSP-C), cause interstitial lung disease (ILD) with pulmonary amyloid deposits. We will discuss the available information describing the biologic and biophysical findings that are apparently involved in the prevention of one form of amyloid, i.e., that formed by the Aβ protein seen in the plaques in human AD, by BRICHOS and TTR, molecules that are direct precursors of other distinct forms of human amyloidosis and what this may mean in the universe of protein-protein interactions in complex organisms.

## BRICHOS structure

**Figure 1:**
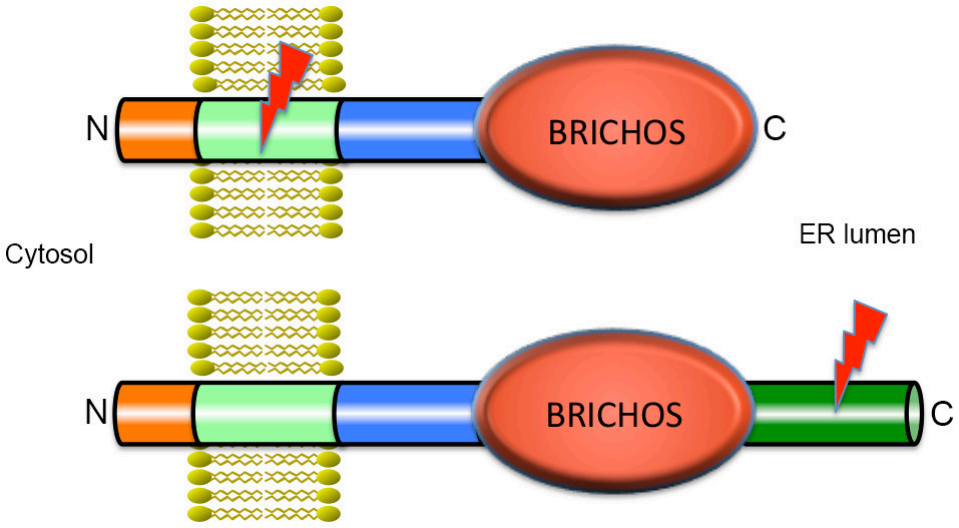
**Architecture of BRICHOS containing proteins**. All proteins that contain a BRICHOS domain have a similar modular structure with a short N-terminal part in the cytosol, a transmembrane (TM) region, a linker region, the BRICHOS domain, and in most cases, a C-terminal region. ProSP-C is depicted in the upper part of the figure and is the only example where the BRICHOS domain is not followed by a C-terminal region. All other BRICHOS containing proteins, depicted in the lower part of the figure, (i.e., the Bri family, chondromodulin, and tenomodulin, gastrokines, and group C, see Hedlund et al., 2009) contain a C-terminal region of varying length. In spite of their similar architectures, only the amino acid sequence of the BRICHOS domain is conserved between BRICHOS containing proteins, while the other parts are structurally unrelated. The lightning symbols denote the regions that are amyloidogenic, the TM region in proSP-C and the C-terminal region in Bri2, see text for details.

The BRICHOS domain is found in several different precursor proteins. In proSP-C and Bri2 these precursors also contain segments that are amyloidogenic (Figure 1). The transmembrane (TM) part of the mature lung surfactant protein C (SP-C) is an archetypical discordant α-helix composed of a long poly-Val segment, i.e., the most β-sheet prone sequence possible (Johansson et al., 1994). As expected from the predicted β-sheet structure, native SP-C can convert into amyloid fibrils *in vitro* (Gustafsson et al., 1999), and expression of the SP-C part only, i.e., without the rest of proSP-C (including the BRICHOS domain) in transgenic mice generates severe SP-C aggregation and toxicity (Conkright et al., 2002). As predicted from the high α-helix propensity of Leu, poly-Val to poly-Leu replacements result in a non-aggregating and functional SP-C analog, (SP-CLeu), which, like SP-C, inserts in surfactant phospholipid membranes, but unlike SP-C, does not form amyloid fibrils, since its α-helical conformation is thermodynamically stable (Kallberg et al., 2001). A synthetic surfactant, presently in clinical trials, has been developed based on synthetic SP-CLeu, (Johansson et al., 2003), demonstrating that modulation of β-sheet and amyloid propensity is a feasible means of designing stable proteins for biologic drug development.

Under physiological conditions, the poly-Val segment in native (pro) SP-C is intrinsically prone to form toxic β-sheet aggregates and amyloid fibrils, and we have proposed that the BRICHOS domain present in proSP-C prevents the poly-Val segment from folding into β-sheet aggregates, and promotes formation of a stable α-helix (Conkright et al., 2002; Johansson et al., 2006). Intriguingly, mutations in the proSP-C BRICHOS domain result in amyloid formation of SP-C and ILD with lung fibrosis, apparently the first described amyloid disease that occurs as result of a mutation in an intramolecular chaperone domain (Willander et al., 2012a).

The BRICHOS domain is found in species ranging from humans to simple marine organisms. It is small (about 100 amino acid residues), has a unique fold and is present in a diverse set of pro-proteins that generate bioactive peptides after proteolytic processing (Hedlund et al., 2009). BRICHOS has been identified in 10 human protein families and the name is derived from BRI2, CHOndromodulin-I and Surfactant protein C (SP-C). The proteins containing a BRICHOS domain have a wide range of functions, and disease associations, including ILD with amyloid deposits (proSP-C), dementia (Bri2), and cancer (Chondromodulin-I) (Sánchez-Pulido et al., 2002; Willander et al., 2012a). There are low pairwise sequence identities between different BRICHOS domain families (~15–25%) but all have similar predicted secondary structures. The precursor proteins have a common overall architecture, and are predicted to be type II TM proteins (Sánchez-Pulido et al., 2002; Hedlund et al., 2009; Knight et al., 2013) with the N-terminus located in the cytosol (Figure 1). All BRICHOS containing proproteins have a cytosolic segment, a hydrophobic TM region, a linker region followed by a BRICHOS domain, and a C-terminal region except proSP-C, in which there is no C-terminal region following the BRICHOS domain. All the proproteins except proSP-C have a segment with high β-sheet propensity, the C-terminal region. In proSP-C the high β-sheet propensity is found in the TM region (Sánchez-Pulido et al., 2002; Hedlund et al., 2009). In all BRICHOS containing pro-proteins the regions that are prone to form β-sheets are well conserved, and are likely to be the BRICHOS client regions that are destined to aggregate in the absence of functional BRICHOS.

**Figure 2:**
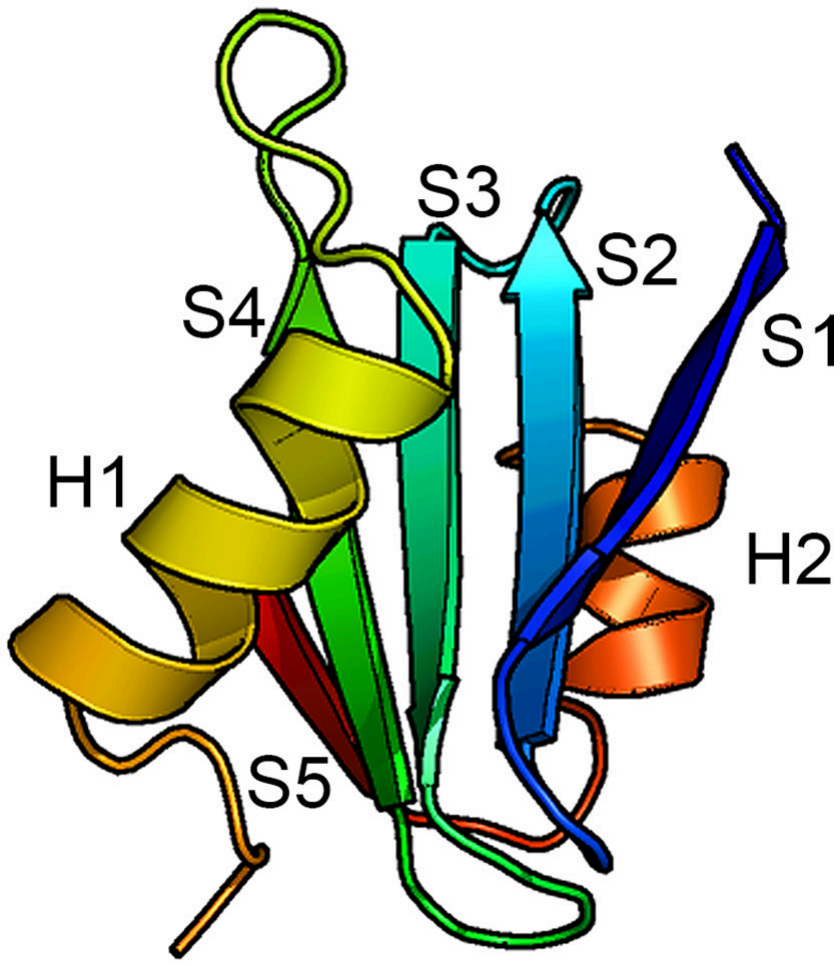
**Structure of the BRICHOS domain**. Backbone conformation of proSP-C BRICHOS (Willander et al., 2012a) with the five β-strands (S1–S5) and the two α-helices (H1 and H2) labeled. Face A of the β-sheet is localized toward H1 and face B is localized toward H2.

The only BRICHOS crystal structure thus far determined is that of proSP-C BRICHOS (pdb code 2yad). It has a unique fold composed of five β-strands arranged in a mixed anti-parallel and parallel fashion, and two flanking α-helices (Figure 2). Helix 1 packs against face A of the β-sheet and helix 2 packs against the opposite side of the β-sheet, face B. Molecular dynamic simulations suggest that helix 1 can translocate exposing the underlying face A of the β-sheet. This implicates face A as the binding site for possible substrates (Willander et al., 2012a), but direct evidence of binding of any peptide to face A has not been found. Homology models of the human BRICHOS domains from each family showed that they are compatible with the proSP-C BRICHOS structure with respect to the secondary structural elements, but the loop regions are highly variable among different BRICHOS domains. An interesting observation was that face A of the proSP-C BRICHOS contains mainly hydrophobic residues, which are apparently complementary to its hydrophobic target sequence—the TM region of proSP-C. Bri2, and Bri3 BRICHOS instead have a face A that contains several residues with charged side-chains, and the proposed Bri2 and Bri3 BRICHOS target sequences, i.e., the respective Bri2 and Bri3 C-terminal regions that are prone to form β-sheets, contain multiple charged residues. This suggests that the properties of face A reflect its binding preferences in the respective BRICHOS domain (Knight et al., 2013). There are only three strictly conserved residues in all BRICHOS domains, two cysteines and one aspartic acid. The cysteines form a disulfide bridge in proSP-C BRICHOS and their strict conservation suggests that a corresponding disulfide bridge is present in all BRICHOS domains.

## Transthyretin (TTR) structure

**Figure 3:**
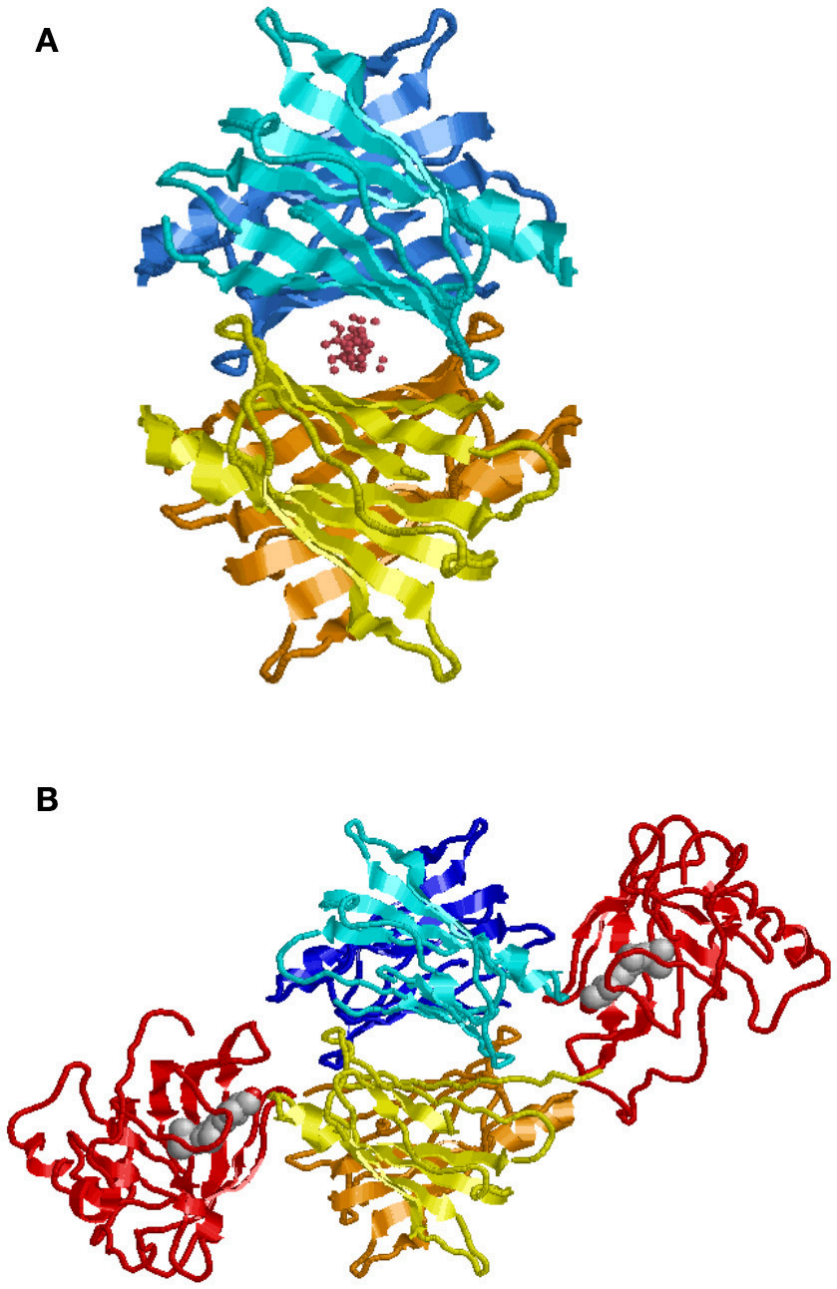
**Model of TTR tetramer**. Shown with ligand in T4 binding pocket **(A)**. Shown with two molecules of retinol binding protein (RBP) charged with retinol **(B)** (Monaco, 2002). While there are two potential T4 binding sites, binding of the first results in an allosteric change restricting access to the second. In humans, generally only one of the RBP binding sites is occupied.

In contrast to BRICHOS, which represents a domain common to a diverse family of proteins, some of which are known to be amyloidogenic, TTR is a unique human protein synthesized in hepatocytes, retinal pigment epithelial cells, choroid plexus epithelium, pancreatic α-cells, Schwann cells, and neurons under some conditions. It is the major carrier of retinol binding protein (RBP) charged with retinol in serum and a minor carrier of the thyroid hormone precursor thyroxine (T4) prior to its conversion to the physiologically more active tri-iodo—thyronine (T3) by tissue deiodinases (Buxbaum, 2007). Only a small fraction of the circulating TTR carries T4 while 25–50% is loaded with RBP-retinol. However, in cerebrospinal fluid choroid plexus synthesized TTR is the major T4 carrier. TTR is a non-disulfide linked homo-tetramer in which the mature polypeptide monomer, after cleavage of the leader sequence, contains 127 amino acids. The tetramer is thermodynamically and kinetically quite stable with a Ka = 1.1 × 10^24^M^−3^(Hurshman et al., 2004). The crystal structure shows a twofold axis of symmetry (Blake et al., 1978). It is assembled as a dimer of dimers around a central channel, which is primarily hydrophobic and contains the two T4 binding sites (Figure 3). T4 binding in the first site induces an allosteric change that makes the second site less accessible to its natural ligand (Neumann et al., 2001). While different portions of the protein bind T4 and RBP, both stabilize the tetrameric structure reducing its tendency to dissociate (White and Kelly, 2001).

## proSP-C BRICHOS in interstitial lung disease

The proSP-C gene (*SFTPC*) is located on chromosome 8 and contains 6 exons encoding a 197 amino acid protein. The protein is expressed exclusively in the secretory pathway of pulmonary alveolar type II cells (Mulugeta and Beers, 2006). Proteolytic cleavage of proSP-C eventually generates the 35-residue SP-C, consisting of an α-helical poly-Val TM region plus an 8-residue N-terminal segment located outside the membrane (Johansson et al., 1994, 1995). The SP-C peptide is secreted as part of lung surfactant, into the alveolar space (Beers et al., 1994; Whitsett and Weaver, 2002). SP-C is unique in that although the primary translation product is a TM protein it is ultimately secreted as a lipophilic, mature peptide (Russo et al., 1999). Mutations in the proSP-C gene lead to ILD, a form of fibrosis (Nogee et al., 2001, 2002; Beers and Mulugeta, 2005; Willander et al., 2012a) with Congophilic deposits containing the mature SP-C segment (Willander et al., 2012a). Both inherited and spontaneous proSP-C mutations have been implicated in ILD (Hamvas, 2006) and a systematic search revealed 91 *SFTPC* disease-causing mutations (Litao et al., 2017). Roughly two thirds of the resulting residue exchanges are localized to the BRICHOS domain, but the most frequent mutation (I73T) is localized in the linker region in between the TM region and BRICHOS. A majority of the ILD associated mutations are located in the linker region or in the BRICHOS domain, and these mutations are proposed to lead to amyloid formation of the SP-C peptide (Willander et al., 2012a). The TM α-helix of SP-C has very high β-sheet propensity, since it is composed of mainly valine residues (Kallberg et al., 2001; Johansson et al., 2010; see above under BRICHOS Structure). It has been hypothesized that proSP-C BRICHOS promotes correct folding and insertion into the membrane of the α-helical TM part of SP-C, preventing the formation of amyloid and ILD (Hedlund et al., 2009; Willander et al., 2012a). Consistent with that notion is the observation that native SP-C isolated from lung surfactant, aggregates into amyloid fibrils *in vitro* that can be visualized by electron microscopy (EM), but co-incubation with proSP-C BRICHOS abrogates SP-C fibril formation (Nerelius et al., 2008).

## Bri2 in familial british and danish dementia

**Figure 4:**
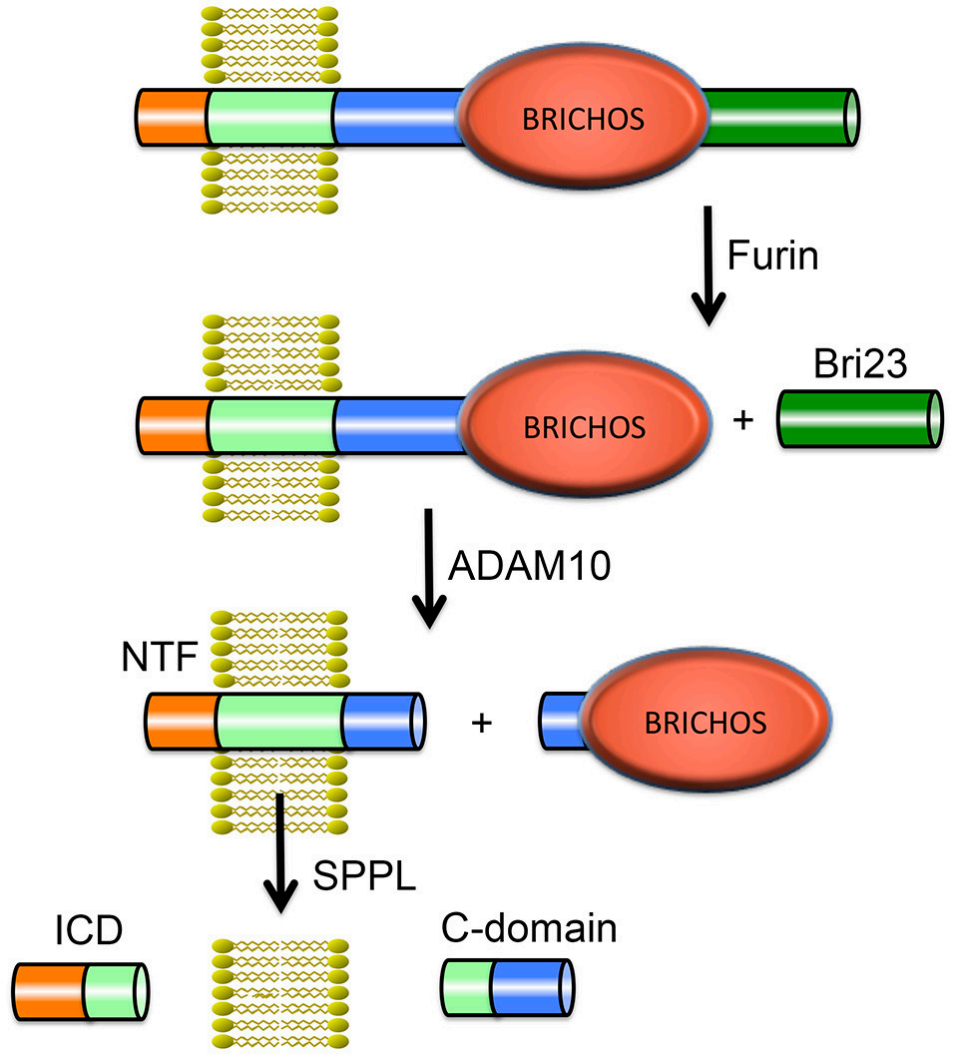
**Proteolytic processing of Bri2**. The full-length Bri2 protein is cleaved by furin resulting in release of the Bri23 peptide, and subsequent cleavage by ADAM10 releases the BRICHOS domain and generates an N-terminal fragment (NTF) that can be cleaved by signal peptide peptidase like (SPPL) proteases into an intracellular domain (ICD) and a released C-domain.

Integral membrane proteins 2B (ITM2B) and 2C (ITM2C) also called Bri2 and Bri3 respectively are part of the BRI family. The Bri2 gene (*ITM2B*) is located on chromosome 13 and contains 6 exons. The Bri3 gene (*ITM2C*) is located on chromosome 2 and contains 7 exons. Bri2 and Bri3 proteins share 42% overall sequence identity, and their BRICHOS domains have 60% identical residues. The BRI family may be the most ancient family of BRICHOS containing proteins, considering it has members in the most ancient species (flies and worms; Sánchez-Pulido et al., 2002). The Bri2 protein is a 266-residue long, type II TM proprotein consisting of an N-terminal cytosolic part (residues 1–54), a TM region (residues 55–75), a linker (residues ~76–130), a BRICHOS domain (residues ~130–231) and a C-terminal region (residues 232–266). Bri2 has an *N*-glycosylation site at asparagine, Asn170 (Tsachaki et al., 2011), and is expressed at high levels in brain, heart, placenta, and pancreas (Vidal et al., 1999). Processing of Bri2 by furin releases a 23-residue peptide referred to as Bri23 (corresponding to residues 244–266 of Bri2) from the C-terminal region. Mutations in Bri2 give rise to release of extended, 34-residue, C-terminal peptides, ABri, or ADan, that deposit primarily in the CNS in two rare amyloid diseases, familial British dementia (FBD) and familial Danish dementia (FDD), respectively (Cantlon et al., 2015a). After the discovery of the pathogenic FBD and FDD mutations and Bri2 as the precursor to the ABri and ADan peptides (Vidal et al., 1999, 2000), furin was identified as the major protease responsible for the proteolytic cleavage releasing the C-terminal peptides (Kim et al., 1999), but other proprotein-like convertases may also process Bri2, releasing C-terminal peptides (Kim et al., 1999; Vidal et al., 2000). Moreover the Bri2 BRICHOS domain can be shed by ADAM10 cleavage and released into the extracellular space, but Bri3 is apparently not processed by ADAM10 (Martin et al., 2008). The remaining membrane associated N-terminal fragment of Bri2, is cleaved by intramembranous proteolysis by signal peptide peptidase-like (SPPL) proteases, SPPL2a and SPPL2b. This cleavage releases a Bri2 intracellular domain (ICD) as well as a secreted so called C-domain (Martin et al., 2008). SPPL cleavage of Bri2 likely takes place secondary to ADAM10 mediated shedding. Moreover ADAM10 processing is not sequence specific but rather occurs at specific distances from the plasma membrane (Sisodia, 1992). Together, the available data are most consistent with ADAM10 releasing the BRICHOS domain by cleavage in the linker region of Bri2. See Figure 4 for an overview of Bri2 processing.

FBD is a rare disease that shares many similarities with AD with memory loss and dementia (Mead et al., 2000). Typical histological findings are amyloid deposition of ABri, cerebral amyloid angiopathy (CAA) and neurofibrillary tangles (NFT's) (Vidal et al., 1999). FDD shares similarities with FBD but patients also show cataracts and deafness. Histological findings in FDD include CAA, NFT's and hippocampal ADan amyloid plaques (Vidal et al., 2000). The FBD pathogenic mutation converts the stop codon in the Bri2 gene to a codon for arginine, extending the open reading frame to include 11 additional amino acids, giving rise to the 34 residue long extended peptide, ABri. The FDD mutation is different and leads to a 10-nucleotide duplication, causing a frame shift replacing the stop codon of Bri2, extending the peptide to another 34 residue peptide, ADan. Both ABri and ADan are thus 11 residues longer than the non-pathogenic Bri23, but the additional residues share no sequence homology (Cantlon et al., 2015b). Aβ has been found in both fibrillar and non-fibrillar deposits of ADan in FDD (Tomidokoro et al., 2005), and Bri2, and/or parts thereof, have been found to deposit with Aβ plaques in AD (Del Campo et al., 2014). These observations suggest possible links between the events underlying the two diseases. It has been suggested that FBD and FDD are caused by the aggregation of ABri and ADan respectively, and/or by a loss-of function of mature Bri2 (Cantlon et al., 2015a). Experimental support for both theories can be found. Data from FBD-Bri2 and FDD-Bri2 knock-in mice as well as human patients show a reduction in Bri2 levels (Tamayev et al., 2010a; Matsuda et al., 2011), and knocking in Bri2 in FDD-Bri2 knock-in mice rescues negative effects on cognition (Tamayev et al., 2010a,b). Moreover, studies show that ABri and ADan aggregation *in vitro* causes cell toxicity (El-Agnaf et al., 2001, 2004) and effects on synaptic plasticity (Cantlon et al., 2015b) similar to Aβ.

## The TTR amyloidoses

TTR is encoded by a single gene on chromosome 18 that encompasses approximately 19 Kb of DNA with 4 exons included within 7 Kb, 6 Kb of upstream (5′) sequence and 6 Kb downstream containing the conventional 3′ non-coding sequence that allows normal mRNA processing after transcription. The promoter proximal 2 Kb appears to contain all the sequences required for tissue specific expression of the gene (Sasaki et al., 1985; Costa et al., 1986; Sparkes et al., 1987; Li et al., 2011; Wang et al., 2014). Despite extensive screening of human populations there have been no reports of a complete absence of a functional TTR protein. However, mouse knockouts survive, are fertile but have a persistent behavioral abnormality with neuronal loss and mild gliosis in the cortex and CA3 region of the hippocampus (Buxbaum et al., 2014).

**Figure 5:**
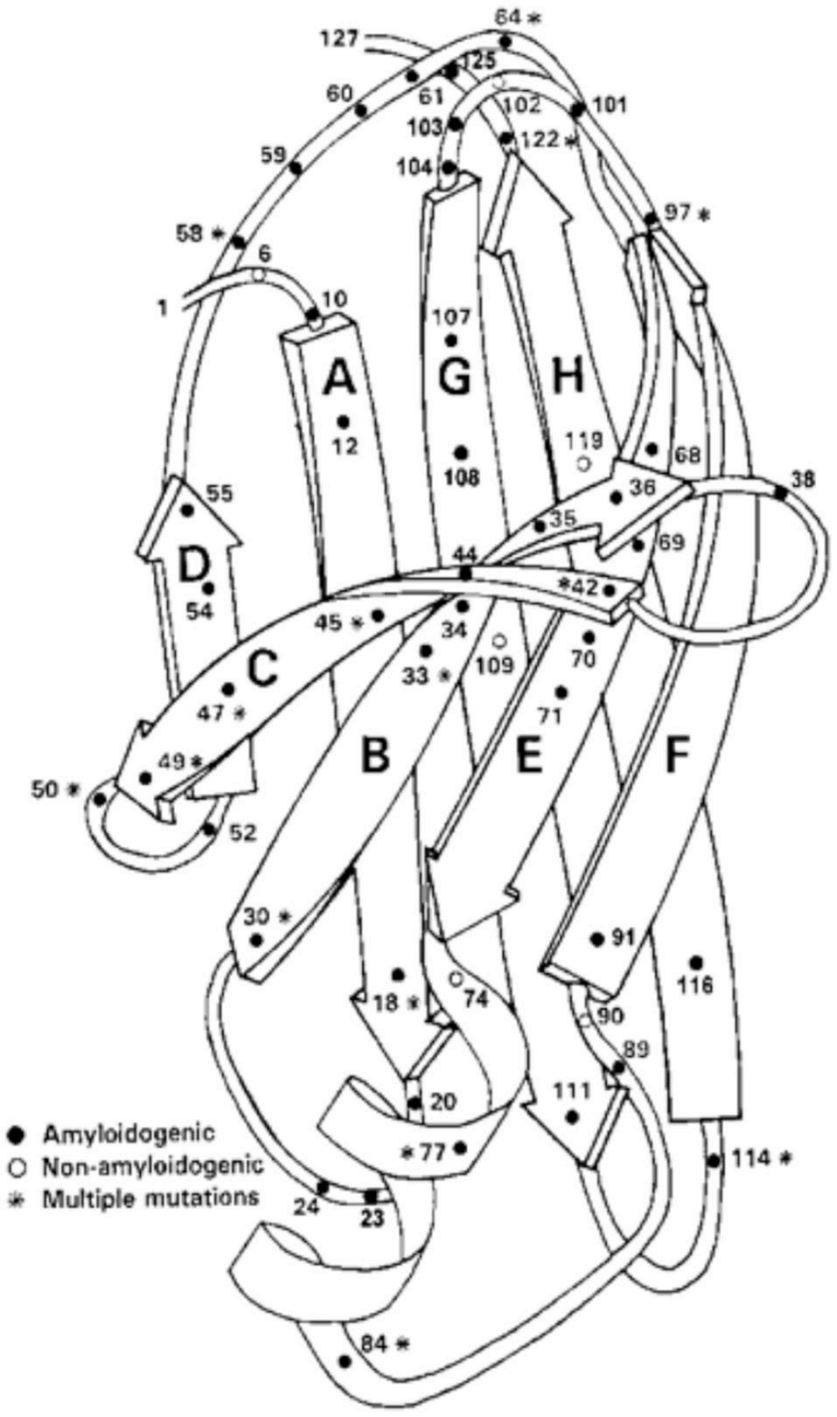
**Ribbon Diagram of TTR Monomer: The figure shows the location of many of the identified amyloidogenic and non-amyloidogenic amino acid substitutions, demonstrating that almost no region of the protein is spared (structure after Blake et al., 1978)**.

Amyloidogenic protein variants causing the autosomal dominant clinical disorders Familial Amyloidotic Polyneuropathy (a sensori-motor and autonomic polyneuropathy) and Familial Amyloidotic Cardiomyopathy have been found in 77 of the 127 amino acids in the protein (Figure 5). Forty residues have been found to have a single amyloidogenic mutation while fifteen have 2, six have 3, five have 4, and one has 5. Fifty amino acids have none and 12 mutations have been described that did not lead to clinically detectable amyloidosis, although two of the involved residues had both amyloidogenic and non-amyloidogenic substitutions (Rowczenio et al., 2014) (Figure 5). There is an increasing frequency of wild type TTR amyloid deposition in the heart, carpal tunnel and gut associated with increasing age currently thought to be related to post-synthetic (perhaps oxidative) changes that may render the wild type protein less stable although other, as yet undefined, mechanisms may be responsible. In the case of the mutations it appears that they all form tetramers which are kinetically or thermodynamically unstable under physiologic conditions resulting in enhanced dissociation releasing monomers which are susceptible to rapid misfolding, aggregation, and fibril formation (Johnson et al., 2012). These observations suggest that the monomers functionally “chaperone” each other.

Recombinant TTR monomers (M-TTR) have been engineered by replacing residues involved in the interaction between monomers required to form the dimers required for tetramerization (F87ML110M) (Jiang et al., 2001). While these monomers can form a functional tetramer they are highly aggregation prone and have provided a useful model to examine the process of aggregation and fibril formation. Such experiments have revealed that aggregation requires the structured monomer to become denatured prior to misfolding and fibril formation (Hurshman et al., 2004). Each monomer contains eight regions of β-sheet, which may explain their inherent tendency to form the β-sheet rich amyloid fibrils. Aggregation appears to primarily involve interaction between the F and H beta strands (Lim et al., 2013). Interestingly murine TTR, which is 80 percent identical to the human protein, is orders of magnitude more kinetically stable and is essentially non-amyloidogenic under physiologic conditions. The crystal structures of the wild type and mutant human proteins and the normal mouse protein are very similar (Hörnberg et al., 2000; Reixach et al., 2008). It is also interesting to note that mouse Aβ and islet amyloid polypeptide are apparently non-amyloidogenic.

Tissue culture studies have shown that exposing target cells to the engineered TTR monomer or amyloidogenic tetramers can induce cytotoxicity. However, the tetramer has to dissociate to release monomer, which appears to bind to cells in a manner consistent with a receptor ligand like interaction, with aggregates forming on the cell surface (within a 3–6 h period) with cell death 48–72 h later (Reixach et al., 2004; Manral and Reixach, 2015). Non-amyloidogenic, non-toxic tetramers are endocytosed directly by the target cells with little evidence of aggregation and no apparent effect on cell viability or function.

*In vivo* it appears that both oligomeric and fibrillar TTR aggregates deposit with an apparent hierarchy of tissue tropism favored by particular mutants. It has been known for some time that limited proteolytic digestion may accelerate amyloid formation by a given precursor and both intact TTR and fragments have been found in the tissue deposits with the relative proportions of each possibly playing a role in the cardiac deposition phenotype (Thylén et al., 1993; Bergström et al., 2005). The mechanism of tissue selectivity, i.e., predominantly peripheral nerve or myocardium, is unclear and may be more apparent than real since at autopsy deposits of fibrils formed from the mutant proteins can be found in most tissues. However, it is possible that different tissues vary in their capacity to digest TTR or in the nature of their extracellular matrix either of which may be related to where fibril deposition occurs.

## BRICHOS interactions with alzheimer's disease peptides and its effects on amyloidogenesis

Bri2 binds AβPP and modulates its processing leading to a reduction of secreted Aβ, both *in vitro* (Fotinopoulou et al., 2005; Matsuda et al., 2005) and *in vivo* (Matsuda et al., 2008; Tamayev et al., 2012), likely because Bri2 restricts the access of the secretases involved in AβPP cleavage, but it has also been shown that Bri2 modulates β-secretase levels (Tsachaki et al., 2013), providing an alternative explanation for how Bri2 affects AβPP processing. Moreover, residues 46–106 (the TM region and parts of the linker) are apparently responsible for binding the juxtamembrane and membrane spanning domains of AβPP (Fotinopoulou et al., 2005), i.e., the BRICHOS domain is not necessary for Bri2 interactions with AβPP in these experimental models (Fotinopoulou et al., 2005; Matsuda et al., 2005). However, an interesting observation is that while the TM region of AβPP is involved in binding to Bri2 (Fotinopoulou et al., 2005), the proSP-C BRICHOS domain and linker region are implicated in correct folding and incorporation of the metastable TM α-helix of SP-C into the membrane (Willander et al., 2012a). It is conceivable that the Bri2 linker together with its BRICHOS domain could have a similar role in incorporating the TM region of AβPP into the membrane. Bri3, which has been less extensively studied than Bri2, is expressed mainly in the brain and co-localizes with AβPP in neurites, and co-immunoprecipitates with BACE1 (β-amyloid converting enzyme 1). Similarly to Bri2, it is cleaved by furin (Wickham et al., 2005). Overexpression of Bri3 reduces cleavage of AβPP, and Bri3 knockdown by RNA interference results in increased levels of Aβ(Kim et al., 1999).

BRICHOS domains exhibit potent anti-amyloidogenic chaperone activity for Aβ peptides and are thought to protect these aggregation prone client peptide sequences from forming amyloid. The two homolog BRICHOS-containing proteins Bri2 and Bri3 are expressed in the human central nervous system (CNS) and are of particular interest in relation to AD. A range of studies suggests that BRICHOS domains can significantly reduce the level of toxic oligomeric amyloid species *in vivo*. For example, co-expression of Aβ_1–42_ and proSP-C or Bri2 BRICHOS in the CNS of transgenic *Drosophila melanogaster* results in delayed Aβ_1–42_ aggregation and dramatic improvements in both lifespan and locomotor function compared to flies expressing Aβ_1–42_ alone (Hermansson et al., 2014; Poska et al., 2016). Results from transgenic mice overexpressing Aβ_1–42_ as a fusion protein with the Bri2 protein support the possibility that BRICHOS prevents Aβ toxicity although it only delays amyloid fibril formation. In this study (Kim et al., 2013), the normal C-terminal peptide of Bri2, Bri23, was substituted for the Aβ_1–42_ sequence, which resulted in generation of free Aβ_1–42_ by proteolytic release. Surprisingly, these mice were not cognitively affected, even though they had high Aβ_1–42_ expression and eventually developed amyloid plaques. The authors suggested that high Aβ_1–42_ levels and aggregates are not sufficient to induce memory dysfunction, and that AβPP processing derivatives (which were obviously not generated from the Bri2-Aβ construct used) are contributing to the toxicity seen in AβPP transgenic mouse models (Kim et al., 2013). However, since the Bri2 BRICHOS domain has been shown to be released from its proprotein by proteolysis ((Martin et al., 2008), Figure 4), an alternative explanation for the lack of toxic effects seen in the Bri2- Aβ_1–42_ expressing mouse model (Kim et al., 2013) could be that liberated BRICHOS domain delays Aβ aggregation and prevents toxicity, in a manner similar to that seen in the fly model. This possibility is further supported by the fact that the Bri2-Aβ expressing mice formed fewer Aβ oligomers than AβPP expressing mice (Kim et al., 2013), a finding which is difficult to explain by the absence of AβPP processing products, but which fits well with the BRICHOS mechanism of action that involves dramatic reduction in oligomer formation (see below).

**Figure 6:**
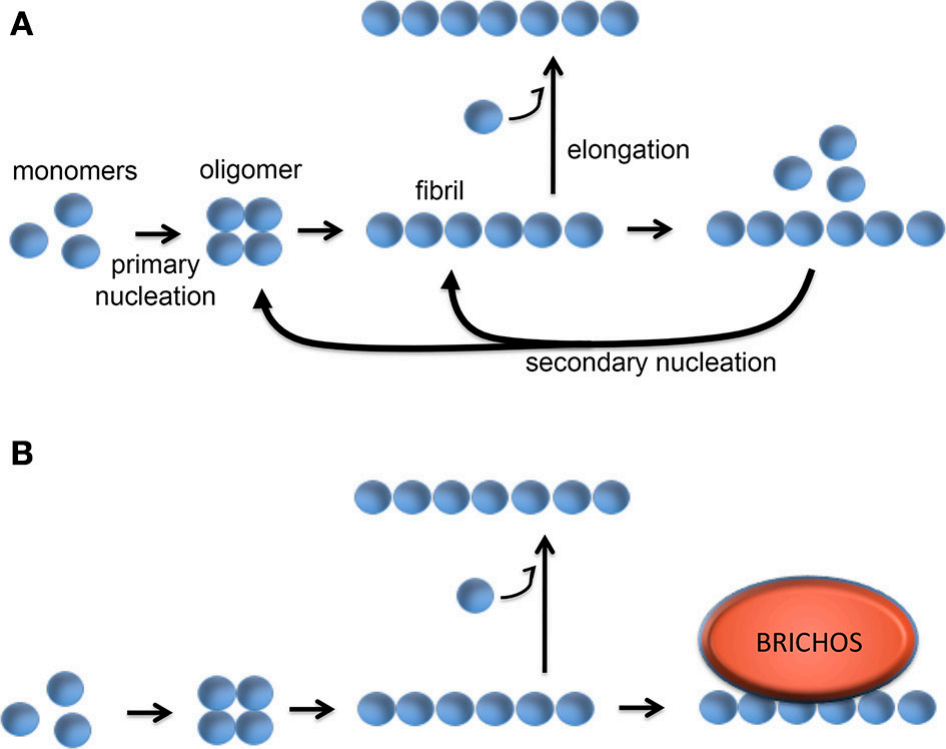
**Molecular mechanism of BRICHOS against Aβ42 fibril formation and toxicity**. **(A)** Aβ42 forms oligomers via a primary nucleation reaction, which thereafter can convert into fibrils. The fibrils can be elongated via addition of monomers, while binding of Aβ monomers to the fibril surface can catalyze formation of oligomers via a secondary nucleation event. **(B)** BRICHOS domain binds to the surface of the Aβ fibrils and thereby markedly reduces the contribution of the secondary nucleation event to the formation of oligomers (Cohen et al., 2015). See text for details.

Studies of the kinetics of *in vitro* Aβ aggregation in the presence or absence of recombinant proSP-C or Bri2 BRICHOS show that sub-stoichiometric amounts of BRICHOS domain significantly slow down fibril formation. It was demonstrated that BRICHOS inhibits the formation of Aβ_1–42_ oligomers by binding to Aβ fibrils and suppressing surface catalyzed secondary nucleation (Cohen et al., 2015; Figure 6). This redirects the reaction pathway toward elongation events, thus minimizing the level of toxic Aβ intermediates. ProSP-C BRICHOS binds to Aβ fibrils with low nM affinity but does not bind to Aβ monomers (Cohen et al., 2015). From the X-ray structure of proSP-C BRICHOS (Figure 2) a mechanism by which BRICHOS domains may prevent amyloid formation by specifically targeting a β-hairpin structure was proposed (Willander et al., 2012a), but it remains to be determined how this mechanism could be applied in the context of binding to fibrils. The detailed mechanism(s) used by proSP-C and Bri2 BRICHOS domains to inhibit Aβ amyloid formation as well as non-fibrillar aggregation apparently differ (see below) and it is possible that BRICHOS exerts different effects toward different client peptides as well as depending on its location, i.e., whether it is intra- or extracellular.

The anti-amyloid activity of BRICHOS extends beyond the physiological client peptides; recombinant BRICHOS from proSP-C and Bri2 efficiently delay fibril formation, and more importantly Aβ_1–42_ toxicity *in vivo* in the CNS of *Drosophila* (Hermansson et al., 2014; Poska et al., 2016). Endogenous Bri2 BRICHOS is found in amyloid plaques in human AD brains (Del Campo et al., 2014), BRICHOS binds with high affinity to Aβ_1–42_ fibrils *in vitro* and this dramatically reduces the formation of toxic Aβ oligomers via a novel and specific mechanism. Taken together, available results suggest that BRICHOS binds to Aβ plaques, and thereby shuts down generation of toxic Aβ species, a mechanism that might be harnessed for AD treatment. The natural expression of proSP-C is restricted to the alveolar epithelium, which makes it an unsuitable to target for treatment of AD and hence ongoing efforts to target AD are mainly focused on the Bri2 BRICHOS, which is expressed in the CNS.

Bri2 is produced in several peripheral tissues and in the brain, with significant expression in neurons of the hippocampus and cerebellum in humans (Vidal et al., 1999; Akiyama et al., 2004). *In vitro*, Bri2 BRICHOS was found to be much more efficient than proSP-C BRICHOS against both Aβ_1–40_ and Aβ_1–42_ fibrillation, and even *in vivo* the Bri2 BRICHOS domain seems to inhibit Aβ_1–42_ toxicity in *Drosophila* central nervous system or eyes more efficiently than pro-SP-C BRICHOS (Willander et al., 2012b; Poska et al., 2016). Bri2 BRICHOS not only blocks the secondary nucleation pathway during Aβ_1–42_ fibrillation, but also affects the elongation process, which could be the reason why Bri2 BRICHOS is more efficient than proSP-C BRICHOS (Arosio et al., 2016). Moreover, proSP-C BRICHOS, like TTR, (*vide infra*) has low “general” molecular chaperone activity (traditionally defined as ability to prevent non-fibrillar aggregation of destabilized model substrate proteins), while Bri2 BRICHOS efficiently suppresses aggregation of destabilized proteins (Poska et al., 2016). Recombinant proSP-C BRICHOS is predominantly a trimer while Bri2 BRICHOS forms mainly large complexes (Poska et al., 2016), and therefore it was suggested that different quaternary structures mediate molecular chaperone and anti-amyloid activities, respectively, analogous to the situation for small heat shock proteins (sHSPs) (Roman et al., 2016). BRICHOS, like many other molecular chaperones, is apparently “stored” in an inactive form in which the binding surface is buried, thus avoiding inadvertent interactions with non-clients. For proSP-C BRICHOS the inactive form is a homotrimer, and consequently, dissociation of the trimer into monomeric subunits should release active BRICHOS. Experimental data support this possibility; addition of low molecular mass ligands increases both the ratio of proSP-C BRICHOS monomer/trimer and the anti-amyloid activity (Biverstål et al., 2015). Bri2, in contrast to proSP-C, BRICHOS forms mainly polydisperse oligomers but also monomers and dimers are found, and it remains to be established which species mediate the ability to prevent Aβ fibril formation and toxicity.

## TTR interactions with Aβ conformers and its effects on *in vivo* and *in vitro* amyloidogenesis

An *in vivo* relationship between TTR and AD was suggested when it was shown that TTR in the CSF could bind Aβ. It was the third CSF protein to display this activity, the other two being ApoE and Clusterin (ApoJ) (Ghiso et al., 1993; Strittmatter et al., 1993; Schwarzman et al., 1994). Early *in vitro* studies indicated that TTR could inhibit the formation of Congo red binding fibrils by Aβ (Schwarzman et al., 2004). *In vivo* studies demonstrated that *C. elegans* transgenic for an Aβ construct driven by the unc-54 muscle promoter displayed abnormalities in motility which were abrogated when they were co-transfected with a TTR cDNA driven by the same promoter (Link, 1995). Tg2576 mice transgenic for a mutant human Aβ gene had increased TTR expression in the cerebral cortex and unilateral injection of an anti-TTR antibody resulted in increased deposition of Aβ on the ipsilateral compared with the contralateral side of injection (Stein et al., 2004). Most definitively, when APP23 AD model mice were crossed with mice over-expressing a wild type human TTR gene the behavioral and neuropathologic features of Aβ deposition were suppressed (Buxbaum et al., 2008). Cortical and hippocampal Aβ_1–40/42_ deposits were reduced by 60–75%. The hyper-phosphorylation of Tau seen in the APP23 mice was diminished and the mice did not develop the defect in spatial learning seen in mice expressing only human Aβ. When the same APP23 mice were crossed with *Ttr* knockout mice amyloid lesions appeared at 4.5 months of age compared with 9 months in wild type APP mice. Similar findings were seen in a different Aβ transgenic strain that were hemizygous for the *Ttr* knockout, only with a somewhat less accelerated phenotype, suggesting a TTR gene dose effect (Choi et al., 2007). The development of pathology did not seem to be accelerated in the absence of *Ttr* in transgenic models that rapidly developed Aβ deposition (Doggui et al., 2010).

In parallel studies it was shown that 70% of cortical and hippocampal neurons in human AD brains (vs. 10% in brains of age matched non-demented subjects) stained with an antibody specific for TTR as did virtually all the cortical and hippocampal neurons in the APP23 mice. TTR was also noted to be present in the Aβ plaques and in vessel walls that contained Aβ deposits. AβTTR-Aβ complexes were isolated from some human AD brains and the brains of the APP23 mice (Li et al., 2011). MRNA analysis of cultured primary neurons from APP23 mice revealed that the TTR staining was a function of increased neuronal synthesis not uptake from the extracellular space (Li et al., 2011). This was the first demonstration of TTR synthesis by primary neurons and that neuronal TTR transcription was increased in the presence of a human AβPP gene. Chromatin immunoprecipitation (ChIP) studies showed that in neurons (in contrast to hepatocytes) TTR is up-regulated by the general stress response regulator Heat Shock Factor 1 (HSF1) which binds to specific sequences in both the human and mouse promoter region (Wang et al., 2014). The parallel increases in transcription of HSP's 40, 70, and 90, supported the notion that TTR behaves as a neuronal stress protein. Detailed molecular studies of CHO cells stably transfected with either wild type or mutant human AβPP genes, then transfected with a human TTR construct, revealed that TTR also bound to the intact APP protein and the C99 β-secretase cleavage product. The latter interaction (between the T4 binding pocket of TTR and amino acids A665, T668, and G659 of C99) resulted in reduced Aβ in the culture medium presumably because TTR binding interfered with the γ-secretase cleavage required for the generation of Aβ either because of an allosteric effect (as suggested for Bri2, *vide supra*) or the reduction in phosphorylation of T668 (Li et al., 2016).

Several laboratories have studied the interaction of TTR with Aβ *in vitro* in attempts to define the biophysical basis of its apparent salutary effect *in vivo*. The earliest studies of the effect of various TTR variants on *in vitro* Aβ aggregation were somewhat difficult to interpret because the only assay utilized was the inhibition of Congo red binding and the puzzling result that some TTR variants actually seemed to enhance Aβ aggregation as measured in this assay (Schwarzman et al., 2004). Since the molecular nature of the starting Aβ material was not stringently analyzed the nature of the interaction could only be hypothesized. Subsequent studies of the inhibition of red blood cell lysis and neuroblastoma cell apoptosis by a highly aggregation prone (but not naturally occurring) sub-fragment of Aβ_25−35_ by TTR also lacked biophysical or structural studies examining the nature of the interaction (Giunta et al., 2005).

The first rigorous biophysical analysis of the interaction utilized TTR isolated from human plasma and a synthetic form of Aβ_1–40_ and showed that TTR sub-stoichiometrically slowed the rate of Aβ aggregation. A mathematical model suggested that TTR both slowed protofibril elongation and the lateral association of protofilaments to produce fibrils. The authors hypothesized that TTR interacted with aggregated rather than monomeric Aβ (Liu and Murphy, 2006). Subsequent studies comparing the effects of recombinant TTR and TTR isolated from plasma were reported as showing that the plasma protein slowed aggregation but did not inhibit cytotoxicity while the recombinant protein increased aggregation and was an effective inhibitor of cytotoxicity. The investigators attributed the differences in activity to the previously described sulfonylation of Cys10 in the plasma protein that was not seen in the recombinant molecule, however there are other possible explanations and a clear explanation is not yet available (Liu et al., 2009).

Other investigators used ^125^I-labeled recombinant TTR to study the interaction between TTR and Aβ_1–42_ in a competition binding assay (Costa et al., 2008a). In contrast to the earlier work they reported that TTR binding was similar with Aβ monomers, oligomers, and fibrils. They also reported that binding of different TTR variant tetramers to Aβ was proportional to the stability of the tetramers, findings that were not consistent with the results of studies from other laboratories examining the capacity of tetramers of different stabilities to inhibit fibril formation, suggesting that binding and the inhibition of aggregation may not be directly related (Du and Murphy, 2010; Li et al., 2013). The same group reported that TTR could behave as a disaggregase of Aβ oligomers and fibrils via its intrinsic cryptic protease activity, an observation that has not been reproduced by others (Costa et al., 2008b; Li et al., 2013).

Several laboratories have shown that recombinant M-TTR bearing mutations (F87ML110M) that do not allow it to form stable tetramers is a very efficient inhibitor of Aβ aggregation, appearing to interact with Aβ oligomers in a sub-stoichiometric fashion. This coupled with the observation that the ability to inhibit Aβ fibril formation was inversely related to tetramer (Du and Murphy, 2010; Li et al., 2013) stability suggested the hypothesis that tetramer dissociation was required for inhibition of Aβ aggregation, a similar scenario has been shown for BRICHOS, for which trimer dissociation into monomers increased the ability to prevent Aβ fibril formation (Biverstål et al., 2015). However, the ability of highly stable variant TTR tetramers such as TTR T119M and TTR K15A and murine TTR (which do not dissociate under the conditions of the *in vitro* experiments) to inhibit fibril formation indicates that tetramer dissociation is not required to inhibit Aβ fibril formation (Li et al., 2013). Further, although the monomer is a more potent inhibitor of fibrillogenesis *in vitro, in vivo* TTR tetramer concentration is one thousand fold that of the monomer hence unless there is a biologic compartment enriched in monomeric TTR and Aβ, it is likely that the tetramer is the active inhibitor *in vivo* (Sekijima et al., 2001, Figure 7).

**Figure 7:**
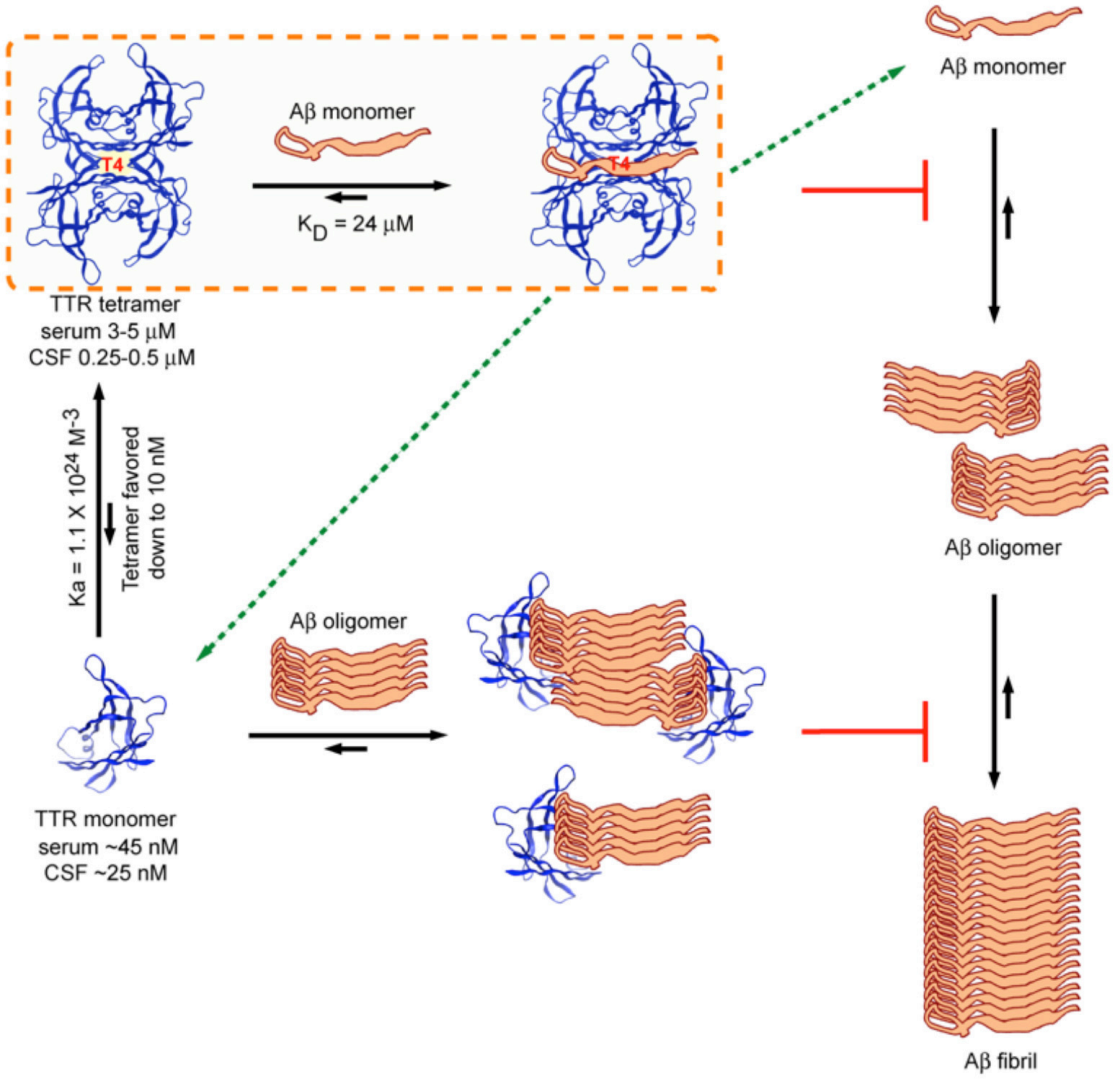
**Model of Interactions between TTR (tetramer) and Aβ_1–40/42_ and M-TTR (engineered monomer) and Aβ_1–40/42_**. The tetramer clearly binds Aβ monomers and pulls them out of the aggregation reaction. It is also capable of binding oligomers and fibrils. M-TTR primarily binds oligomers. What is not clearly shown here is that it is likely that M-TTR is in an oligomeric state when it engages the Aβ oligomers (from Li et al., 2013).

Cross-linking and alanine scanning mutagenesis suggested that TTR strand G and the EF helix and amino acids L17, L82, S85, and L110 were directly involved in Aβ binding (Liu et al., 2009; Du et al., 2012). More detailed precise analysis of binding using nuclear magnetic resonance spectrometry confirmed the involvement of amino acids L17 and L110 but not L82 and S85 (Li et al., 2013). Almost 20 amino acids showed resonance shifts when TTR interacted with the Aβ monomer all located in and around the T4 binding site, suggesting that this region behaves as a predominantly hydrophobic pocket reactive with small T4-like ligands and hydrophobic stretches of some proteins (Li et al., 2013). These data were reinforced by experiments showing that tafamidis, a resveratrol related molecule with high affinity for the T4 binding site, inhibited the inhibition of fibril formation by TTR tetramers but had no effect on the inhibition of fibrillogenesis by the engineered monomeric TTR, which does not form a T4 binding pocket (Johnson et al., 2012).

Isothermal titration calorimetric (ITC) analysis of the interaction between WT TTR tetramer and the Aβ_1–40_ monomer (controlled for the possible self-aggregation of the reactants) had a K_D_ in the micromolar range and a stoichiometry of 1, differing considerably from those reported using other techniques (Li et al., 2013). Interestingly the stoichiometry and K_D_ of the interaction between the human and mouse tetramers and the Aβ monomer were very similar even though the human protein was a much better inhibitor of fibrillogenesis, suggesting, as did other data, that binding and inhibition of fibril formation are not equivalent. The stoichiometry of interaction between tetrameric human TTR and Aβ monomers (approximately 1:1) was much higher than that determined for the inhibition of fibril formation by TTR tetramers (sub-stoichiometric, 1:40–1:100) indicating that in the latter experiments it was likely that TTR also interacted with oligomeric species (Figure 7).

The TTR binding site of Aβ involved the 4G8 epitope, i.e., amino acids 17–21 (Li et al., 2013). Based on western blots, which show interaction of oligomeric M-TTR with Aβ tetramers and octamers, NMR, which showed no resonance shifts on MTTR-Aβ monomer interaction and ITC, which did not detect heat gain or release on incubation of MTTR with monomeric Aβ, the interaction of the recombinant monomeric MTTR appears to require oligomerization of the monomer prior to an interaction with Aβ oligomers rather than the Aβ monomer in a sub-stoichiometric fashion. Similar observations have been made examining the interaction between M-TTR and Aβ_1–42_ using fluorescence correlation spectroscopy (Verghese et al., 2013). It is possible that some of the discrepancies in results between the early studies and the most recent work with respect to the efficacy of inhibition of Aβ by the various mutant TTR tetramers reflects the presence of variable amounts of TTR oligomers formed from monomers generated during the period of incubation of Aβ with different TTR preparations. It was also quite evident that binding studies using conformers bound to fixed surfaces gave different results than experiments performed in liquid phase, e.g., M-TTR bound Aβ_1–42_ monomers fixed to nitrocellulose but no interaction was seen in the liquid phase NMR or ITC experiments (Li et al., 2013).

A series of experiments examining the mechanism of inhibition of Aβ_1–40/42_ cytotoxicity by TTR using both pre-formed Aβ_42_ and HypF-N (a model amyloidogenic bacterial protein that behaves similarly to Aβ_42_) cytotoxic aggregates compared the inhibitory effects of various TTR conformers and showed that M-TTR was the best with human tetramer being less effective and the murine tetramer the least inhibitory, a rank order similar to that seen with inhibition of fibril formation as an assay (Cascella et al., 2013). More interesting was the demonstration that the various TTR species all worked by interacting with the toxic HypF-N oligomeric aggregates to make them larger and less toxic. In a recent follow up analysis the inhibitory process utilized by M-TTR was compared with that seen in the inhibition of HypF-N cytotoxicity by the extracellular chaperone clusterin and the small heat shock protein αB-crystallin (Cappelli et al., 2016). Like the chaperones M-TTR increased oligomer size and reduced the structural order of the aggregates. These data with HypF-N and correlation spectroscopy results with Aβ_1–42_ strongly suggest that M-TTR does not inhibit aggregation, rather it changes the nature of the aggregates allowing them to supersaturate the solution forming non-fibrillar, non-toxic structures. Interestingly while clusterin and the other “extracellular chaperones” haptoglobin and α_2_ macroglobulin also inhibit non-fibrillar protein aggregation, TTR (like proSP-C BRICHOS *vide supra*) only inhibits amyloidogenesis. The mechanism of the latter may reflect the fact that fibrillogenesis requires precise homotypic alignment of β-strand structures (“stearic zippers”) (Goldschmidt et al., 2010). It is possible that while pre-amyloid oligomers form internally homotypic aggregates, those formed by one precursor, e.g., TTR, are not precisely in register with those of the second, i.e., Aβ, perhaps forming “hetero-zippers” (Eisenberg, D, personal communication). The resulting heterotypic aggregates are less structured and incapable of attaining the order required for protofibril formation. This would be different in the case of pairs of amyloid precursors in which cross-seeding occurs in which the heteromeric oligomeric fit does not disrupt the parallel or anti-parallel β-sheet structures required for protofibril formation (Solomon et al., 2007; Oskarsson et al., 2015).

In summary tetrameric human TTR appears to inhibit Aβ aggregation by binding Aβ monomers and removing them from the fibril forming (elongation) pool. It also binds oligomers and fibrils contributing to the formation of larger, non-cytotoxic amorphous aggregates. Since the tetramer is far and away the most prevalent form of TTR *in vivo* it is likely that this represents a significant component of its protective effect in the mouse models. What happens to the TTR-Aβ complexes is unclear, perhaps as hypothesized elsewhere, it enhances Aβ transport out of the brain (Alemi et al., 2016). The recent data that it is also capable of decreasing Aβ production (at least in cultured cells) suggests a possible additional mechanism.

While monomeric TTR may not be functionally protective *in vivo* the comparison of the biophysics of its interaction with Aβ and HypF-N with that seen in molecules long known to be members of the proteostatic network, i.e., clusterin and αB-crystallin has given some insight into how those molecules may function *in vivo*.

## Fighting fire with fire: do amyloid precursors have therapeutic potential in AD?

From the forgoing it is evident that BRICHOS domains and TTR tetramers and monomers can inhibit Aβ oligomerization, fibril formation and cytotoxicity *in vitro*. It also appears that genetically induced over-expression of the two parent molecules can suppress or delay the eventual development of the neuropathologic and behavioral abnormalities seen in transgenic fly or mouse models of human Aβ deposition (Table 1). At this moment such genetic approaches are not possible in human AD. However, it is likely for TTR at least, that human neurons may already be utilizing this molecule as a defender of neuronal integrity in the face of proteotoxic and perhaps other forms of cellular stress. As noted above, the majority (70%) of neurons in human AD brains make TTR, compared with 10% in age matched non-demented control brains, a finding also seen in transgenic models of human Aβ deposition. Further both TTR and Bri2 have been found in human AD plaques. The latter may reflect either that they are behaving as co-conspirators or more likely is that those molecules represent failed (or successful, if the fibrils are the least toxic form of Aβ aggregates) chaperones. These observations pose the question does AD represent an age related failure of host neuronal defense mechanisms? If that is the case can the failure be overcome therapeutically? While current approaches to AD focus on reducing Aβ production, might we do better by enhancing host resistance or some combination of the two?

**Table 1:** **Alzheimer's disease related activities of Transthyretin and BRICHOS—containing proteins**.

**Feature**	**Transthyretin (TTR)**	**BRICHOS (Bri2)**
Clinical amyloidosis human	FAP, FAC, SSA	FBD, FDD
Amyloid formation *in vitro*	+	+
Protein topology	Unique Homotetramer	Domain in many proteins
Interaction with Aβ *in vitro*	Monomers, oligomers, fibrils	+
Transgene interaction with AD model *in vivo*	suppresses	suppresses
Effect of knockout on AD model	accelerates	?
Presence human AD plaques	+	+
Effect of knockout on CNS function (no AD gene)	Behavioral abnormality	?
Increased neuronal synthesis AD	+	?
Inhibits primary nucleation Aβ	+	−
Inhibits secondary nucleation Aβ	?	+
Inhibits elongation Aβ	+	+

### Gene therapy

Direct administration of a *TTR* or BRICHOS containing gene to humans is currently not feasible, however it may become possible to isolate totipotent stem cells from subjects with AD, differentiate them to neurons or astrocytes, engineer them to contain a wild type human TTR or BRICHOS containing gene regulated by either their own or an inducible promoter and administer those cells to the patient from whom they were obtained. Currently there are many unknowns surrounding this approach. It assumes that a mode of administration will be available to insure that the cells reach the nervous system and home to regions of pathology. The gene must include all the regulatory sequences required for tissue specific expression and protein production in the secretory pathway so that the encoded protein will be available to interact with Aβ and its oligomers secreted by the endogenous neurons. If the gene is driven by an inducible promoter the inducing agent must be able to cross the blood brain barrier. Further the long term behavior of cells differentiated from pluripotential precursors is currently unclear.

### Protein or peptide therapy

A large number of proteins including TTR and BRICHOS and peptides derived from them have been shown to inhibit Aβ aggregation *in vitro* (e.g., Mangrolia et al., 2016). Currently it is difficult to deliver these molecules to the central nervous system, although there have been some successes administering molecules such as insulin by nasal spray (Hölscher, 2014).

One way to circumvent the problems inherent in protein or peptide delivery has been to use Adenoviral, AAV or lentiviral vectors as carriers for sequences encoding the therapeutic protein or peptide (e.g., Kim et al., 2004; Bourdenx et al., 2014; Parr-Brownlie et al., 2015; Blessing and Déglon, 2016; Saraiva et al., 2016). Vectors have been developed that will preferentially target neurons although most still require intracerebral inoculation. This approach may be more appropriate for a BRICHOS based reagent than for a TTR related molecule since the quaternary structural demands of the TTR tetramer may be constraining with respect to having excess monomers available to misfold and aggregate rather than interact with some form of Aβ.

A third approach being explored is based on the observation that both systemic and neuronal TTR production go down with increasing age and the assumption that maintaining neuronal TTR production throughout life will continue to make TTR available to bind Aβ and its soluble aggregates in the environment in which they appear to be neurotoxic. If small molecules can be identified which specifically induce neuronal TTR (or BRICHOS- containing domain) synthesis and can cross the blood brain barrier after systemic administration, they might be able to slow or arrest the progression of neurodegeneration.

All of these approaches present practical problems with respect to delivery, specificity of the cellular and molecular targets and intrinsic amyloidogenicity of the therapeutic agent. In the case of small molecule therapeutics the potential for off-target or mechanism related toxicities is always an issue that cannot be ignored. The delivery and specificity issues are no different than they are for any therapeutic biological. The intrinsic amyloidogenicity of molecules as TTR or BRICHOS-containing proteins, whether encoded by a naked gene, produced by an engineered differentiated pluripotent stem cell or induced by a small molecule is a real risk. It would not be good to either enhance amyloid oligomer formation by endogenously produced Aβ or induce the synthesis of a sufficient amount of the therapeutic anti-amyloid to exceed the critical concentration required to nucleate its own fibrillogenesis. Based on observations in mice carrying many copies of the wild type human *TTR* gene with all the known elements required for tissue specific expression, it appears that neurons regulate TTR production quite tightly, perhaps precluding the possibility of local TTR aggregation and oligomer formation even while systemic amyloid deposition of liver synthesized TTR goes on (Buxbaum et al., 2008).

In recent years much has been made of the phenomenon of “templated misfolding” as a mechanism for spreading of both Parkinson's and Alzheimer's diseases (Walker et al., 2006; Kordower et al., 2008). It has also been possible to “seed” Aβ aggregation in mice and rats by the intracerebral or parenteral administration of homologous brain or fibril fragments (Brouillette et al., 2012; Heilbronner et al., 2013). In at least one instance the seeding has been inhibited by pre-incubation with human TTR (Brouillette et al., 2012).

In other systems it has also been possible to nucleate murine AA amyloid *in vivo* by the administration of other amyloid aggregates raising the notion of cross species seeding by the ingestion of Foie gras (Solomon et al., 2007). We have described two examples in which two discrete human amyloid precursors rather than “cross seeding” inhibit the formation of cytotoxic Aβ aggregates and fibrils *in vitro* and *in vivo* in transgenic murine models of human Aβ deposition. Further human data suggest that the production of these molecules is increased in the course of human AD, perhaps in the context of neuronal defense. We have reviewed the structural features of these molecules that appear to be responsible for the “protective” heterotypic interactions that prevent the homotypic formation of toxic oligomers and fibrils *in vivo* and speculate that these interactions may not be coincidental but represent an evolutionarily conserved mode of neuroprotection.

## Author contributions

JJ wrote the sections on the BRICHOS proteins. JB wrote the sections on transthyretin. Both contributed to the introduction and the potential therapeutics sections and both edited and reviewed the entire manuscript.

## Funding

Work from the JJ laboratory was supported by the Swedish Research Council.

### Conflict of interest statement

JB has been a paid consultant for Foldrx, now Pfizer, Alnylam, Ionis (formerly Isis) and Prothena Pharmaceuticals, all of whom have therapies in development for the transthyretin amyloidoses, none of whom have contributed materially to the work described herein. The other author declares that the research was conducted in the absence of any commercial or financial relationships that could be construed as a potential conflict of interest.
